# Factors associated with 25-year kidney allograft survival: a single-center retrospective analysis

**DOI:** 10.3389/fmed.2026.1745986

**Published:** 2026-02-25

**Authors:** Anna Anyszek, Łukasz Czyżewski, Magdalena Durlik, Janusz Wyzgał

**Affiliations:** 1 Department of Nephrology Nursing, Medical University of Warsaw, Warsaw, Poland; 2 Department of Geriatric Nursing, Medical University of Warsaw, Warsaw, Poland; 3 Department of Transplantation Medicine, Nephrology and Internal Diseases, Medical University of Warsaw, Warsaw, Poland

**Keywords:** acute rejection, cold ischemia time, creatinine, early gray failure, hypertension, kidney transplantation, ultra-long graft survival

## Abstract

**Introduction:**

One-year outcomes after kidney transplantation (KTx) have improved markedly; however, ultra-long graft survival remains rare. We analyzed an outcome-defined sample from a single-center, single-era cohort to identify early, potentially modifiable factors associated with ≥25-year graft survival and to translate these associations into pragmatic, hypothesis-generating, center-level process targets.

**Methods:**

We retrospectively reviewed the first adult solitary KTx performed in 1980–1995. Two outcome-defined, unmatched groups were compared: ultra-long survivors (ULS; graft survival ≥25 years; *n* = 59) and early graft failure (EGF; ≤10 years; *n* = 61). We extracted pre- and peri-transplant variables, first-year rejection burden (0/1/≥2 treatment cycles), and longitudinal laboratory data. Unconditional logistic regression was used to estimate adjusted associations (odds ratios [ORs]) in a complete-case subset with a pre-specified, parsimonious adjustment (recipient and donor age).

**Results:**

Cold ischemia time (CIT) was shorter in ULS than in EGF (1281.8 ± 473.9 vs. 1764.8 ± 564.9 min; *p* = 0.016; mean difference 483 min) and was associated with higher odds of EGF (per 60 min, adjusted OR 1.29; 95% confidence interval (CI) 1.02–1.63; *p* = 0.032). ULS had a lower first-year rejection-treatment burden (≥1 antirejection treatment cycle, 40.7% vs. 63.9%; *p* = 0.017). Early kidney function profiles favored ULS (lower creatinine at 6 months, 1 year, and 5 years; all *p* ≤ 0.004; higher hemoglobin at 5 years; *p* < 0.001). Exploratory time-to-event analyses showed concordant directions for CIT and rejection-treatment burden. In an exploratory univariable landmark analysis, 6-month creatinine levels showed moderate within-sample discrimination for EGF versus ULS (apparent area under the curve [AUC], 0.739)

**Discussion:**

Overall, CIT showed the most consistent and potentially actionable association with long-term outcomes in this historical cohort; however, inferences are observational, and residual confounding cannot be excluded.

## Introduction

Chronic kidney disease (CKD) is a common multifactorial condition with a rising global prevalence. Current estimates suggest that about 850 million people worldwide live with kidney disease, underscoring its public health burden (1). In Poland, recent epidemiological data indicate that up to 4 million individuals have CKD, most of whom are undiagnosed, highlighting registry under-ascertainment and the need for system-level action (2, 3). The large population potentially requiring renal replacement therapy (RRT) makes it essential to optimize survival and quality of life. Kidney transplantation (KTx) remains the preferred treatment for end-stage renal disease based on its clinical benefits, economic considerations, and superior health-related quality of life compared with other RRT modalities. It is also the only RRT that restores endocrine kidney function (4, 5). Although short-term outcomes after transplantation have improved substantially, long-term graft survival, particularly death-censored survival, has modestly improved beyond the first decade. This has kept attention on ultra-long survivors and on the early, potentially modifiable determinants of late outcomes. Here, we examine pre- and peri-transplant factors associated with ≥25-year graft survival and consider how these signals could inform clinical quality improvement. Cohorts explicitly focusing on ultra-long survivors remain scarce; available ≥20–25-year reports are mostly single-center, use heterogeneous definitions, and are often underpowered for multivariable analyses (6, 7). Cold ischemia time (CIT) is one of the few clearly modifiable center-level determinants of transplant outcomes. Contemporary studies link a longer CIT to early complications such as delayed graft function (DGF) and, indirectly, to worse longer-term performance. In current practice, strategies such as hypothermic machine perfusion may mitigate risk when the CIT is prolonged (8). However, evidence linking pragmatic, center-level CIT targets to ≥25-year survival remains limited, which is a practical gap that our study addresses (6). Early immunologic injury is central: first-year biopsy-proven rejection (clinical or subclinical) is repeatedly associated with worse long-term trajectories, although granular, time-varying modeling of the rejection burden is rarely feasible in historical datasets. Recent multicenter work also indicates that the nature and timing of rejection episodes shape later risk (9). Cardiometabolic comorbidities are common after KTx. Hypertension affects approximately 70–80% of recipients at 1 year and is linked to cardiovascular disease, the dominant cause of late morbidity and mortality; however, optimal blood pressure targets and their translation into improved graft survival remain debated (10). Similarly, post-transplant diabetes mellitus (PTDM) is associated with worse patient and graft outcomes, supporting proactive screening and immunosuppression choices that consider glycemic risk (11). Simple early functional markers (e.g., 6–12-month serum creatinine) retain prognostic value and can serve as actionable waypoints; however, most studies focus on 3–5-year horizons rather than ≥25-year outcomes (12, 13). Accordingly, in a single-center cohort transplanted in 1980–1995, we assess whether early, potentially modifiable signals (CIT, first-year rejection burden, and early kidney function profiles) are enriched among recipients with ≥25-year graft survival and can be translated into auditable, center-level quality metrics (7).

The aim of this study was to delineate early and potentially modifiable factors associated with ultra-long kidney allograft survival (≥25 years) versus early graft failure (≤10 years) in a cohort transplanted in 1980–1995. We also sought to translate these associational signals—particularly CIT, first-year rejection burden, and early biochemical profiles—into explicit, auditable, hypothesis-generating targets for clinical practice.

## Materials and methods

This manuscript constitutes the third, analytical stage of a coherent research program conducted in a single center and within a single transplantation era (1980–1995). Stage I (14) was a descriptive study of a cohort of 45 recipients with well-functioning grafts ≥25 years post-transplant, without a comparator arm, focusing on associations between kidney function parameters and hemoglobin (HGB)/creatinine 10 years after KTx. Stage II (15) expanded this characterization to 59 ultra-long-term graft survivors (ULS) and reported detailed immunosuppression and long-term course without a control arm. Stage III does not repeat descriptive results; it introduces, for the first time, a designed comparison between the ULS cohort and a control arm with early graft failure (EGF), performs hypothesis-driven testing, and applies multivariable models—qualitatively distinguishing it from earlier publications and justifying a separate report.

### Study design and setting

This retrospective study was nested within a historical single-center cohort and was conducted at the Nephrology–Transplantation Outpatient Clinic, Department of Transplantation Medicine, Nephrology, and Internal Diseases, Medical University of Warsaw, Infant Jesus Clinical Hospital (Warsaw, Poland). We used an outcome-enriched sampling strategy to compare recipients at opposite ends of the graft survival distribution. The source population comprised adults who underwent their first solitary kidney transplantation at this center between 1980 and 1995 and were followed longitudinally in the institutional transplant clinic. Restricting inclusion to a single era was intended to limit confounding by secular changes in surgical practice, organ preservation, and post-transplant care. All participants were Polish nationals. The study was conducted in accordance with the Declaration of Helsinki and approved by the Bioethics Committee of the Medical University of Warsaw (protocol code: AKBE/167/2022; approval date: 13 June 2022).

### Participants

Two outcome-defined cohorts were analyzed: ULS, defined as graft survival of ≥25 years (*n* = 59), and EGF, defined as graft survival of ≤10 years culminating in hemodialysis (HD) or death (*n* = 61). Eligibility required age of ≥18 years at KTx, transplantation at the study center within the specified era, and documentation sufficient to assign the outcome group and extract core perioperative and follow-up variables. Patients with multi-organ or repeat transplantation, or with records insufficient for outcome classification, were excluded.

The ULS cohort in the present study originated from the same center and era as the previously described groups; a partial overlap of individuals with the 2020 and 2025 reports is possible and expected. However, (1) those earlier studies included only recipients with very favorable long-term outcomes (no EGF arm) and were descriptive/exploratory; (2) the current study defines and incorporates a formal EGF comparator arm, broadens the comparative variable set (including HLA matching, ischemia times, early postoperative parameters, first-year rejection events, and standardized longitudinal electrolyte/hematologic profiles), and applies hypothesis testing with multivariable logistic regression and confidence intervals. All analyses and tables are new relative to the 2020/2025 publications and address distinct, comparative research questions. The number of ULS is fixed by definition and era. To maximize statistical information, the comparator cohort was assembled as inclusively as feasible within the same center and time period, while requiring a minimal set of extractable core peri-transplant variables for analysis. Further expansion of the comparator arm was limited by incomplete covariate capture. Accordingly, we prioritized (1) inclusion of all eligible EGF cases with sufficient core data, (2) pre-specified parsimonious adjustment to mitigate overfitting, and (3) consistency of findings across complementary analytic approaches. No individual or frequency matching was performed, and cohorts were defined solely by outcomes within the same center and transplantation era. We screened all eligible EGF cases from the same era at our center; all cases meeting the minimal documentation requirements for core covariates were included, and further expansion was limited by archival covariate completeness.

### Variables

Clinical data were extracted from institutional medical records using a standardized template. Recipient variables included age and sex at KTx, primary kidney disease (biopsy-confirmed when available), pre-transplant RRT exposure (hemodialysis [HD] and peritoneal dialysis [PD] durations), hypertension status before and after KTx, and hepatitis B status (pre-existing and acquired). Early postoperative course variables included acute tubular necrosis, need and duration of post-transplant dialysis, and index hospitalization length. Donor and procedural variables included donor age and sex, ABO and Rh compatibility, HLA matching, graft laterality, implantation site (left/right iliac fossa), and ischemia metrics (warm ischemia time, CIT, and total ischemia time). Longitudinal laboratory measurements (HGB, white blood cell count, platelet count, serum creatinine, potassium, and sodium) were recorded at pre-specified intervals: early postoperative period, 1 and 6 months, 1 year, and every 5 years thereafter, while the graft functioned. First-year acute rejection was ascertained from inpatient and outpatient records. A recognized rejection episode was defined as any event within 365 days post-transplant meeting at least one of the following criteria: (1) an explicit clinician-documented diagnosis of acute rejection in the chart; and/or (2) a biopsy report interpreted as acute rejection (when available); and/or (3) initiation of antirejection therapy for suspected/diagnosed rejection (e.g., steroid pulses and/or lymphocyte-depleting therapy) as recorded in the medical record. Because protocol biopsies were not performed and subclinical rejection was not systematically assessed in this historical cohort, events without clinical recognition and/or documentation could not be captured. Accordingly, the absence of a recognized rejection indicates no clinician-documented rejection diagnosis, no biopsy interpreted as rejection, and no recorded antirejection therapy within the first post-transplant year.

### Group definitions

The ULS group was defined *a priori* as recipients with ≥25 years of functioning graft survival. This threshold was chosen to capture an extreme, clinically meaningful phenotype that has been used in prior single-center series of very late outcomes and long-lifespan allografts and to align with studies that explicitly analyzed survivors in the ≥20–25-year range as a distinct group with different late-phase dynamics (16, 17). EGF was defined as the loss of graft function or return to dialysis within ≤10 years post-transplant. The 10-year boundary reflects a decennial clinical benchmark widely used in transplant reporting, concentrates the majority of non-survivors in a period dominated by perioperative and early immunologic mechanisms, and helps yield a comparator group of similar size within this single-era dataset (18, 19). To avoid phenotype mixing, patients with survival >10 and <25 years were excluded from the primary contrast; sensitivity checks with neighboring cutoffs (≤8 and ≤12 years) produced directionally consistent associations. This design intentionally focuses on outcome-defined groups at the tails of the survival distribution to enhance signal detection, rather than to model the full spectrum of post-transplant trajectories.

The time of origin was the date of KTx. For descriptive contrasts, patients were censored at 10 years or the last follow-up, whichever came first. Death with a functioning graft was treated as a competing event in the Fine–Gray models. Because of the low event counts, first-year rejection was summarized as a categorical burden (0/1/≥2 treatment cycles) rather than modeled as a fully time-varying covariate. CIT was analyzed in minutes, with effect estimates reported per 60-min increment for interpretability; clinical targets are expressed in hours where relevant.

### Outcomes

The primary endpoint contrasted EGF (≤10 years) versus ULS (≥25 years). Secondary rejection-related outcomes were derived from the above ascertainment algorithm and included the following: (1) presence/absence of recognized rejection within 1 year, (2) number of recognized rejection treatment cycles (0/1/≥2), and (3) days of antirejection therapy within 1 year.

### Statistical analysis

The significance level was set at *p* < 0.05. All statistical analyses were performed using Statistica 13.3 (TIBCO Software Inc., California, United States). Continuous variables are reported as mean (SD). For bounded variables that were right-skewed and/or zero-inflated, and to avoid over-interpretation of mean±SD notation, observed ranges (min–max) were additionally provided when SD exceeded the mean. For univariate comparisons, categorical variables were analyzed using Fisher’s exact test (extended Fisher or chi-square, where appropriate, depending on table size and expected counts), and continuous/ordinal variables were compared using the Mann–Whitney U test (Wilcoxon rank-sum), unless assumptions for a two-sample *t*-test were met. Normality was assessed using the Shapiro–Wilk test, and homogeneity of variances was assessed using Levene’s test. When both were acceptable, Student’s *t*-test was applied. All tests were two-sided.

Unconditional logistic regression was used to model the odds of belonging to the EGF (≤10 years) versus the ULS (≥25 years) group, as no individual or frequency matching was performed; the results are reported as odds ratios (ORs) with 95% confidence intervals. As this is an outcome-enriched extreme-group sample, ORs quantify associations with the odds of group membership in this dataset and should not be interpreted as population-level risks or absolute probabilities in an unselected transplant population. Primary models (e.g., adjusted CIT analyses) were not FDR-adjusted. Missing data were handled using complete-case analysis; denominators, therefore, vary across variables due to incomplete archival documentation. Model specification and covariate adjustment were pre-specified and intentionally parsimonious, given the limited effective sample in complete cases; consequently, conclusions emphasize effect sizes with a note of uncertainty rather than dichotomous significance. Since the number of ULS is fixed by definition and era, statistical efficiency was maximized by (1) constructing a maximally inclusive EGF comparator arm, (2) avoiding variable selection procedures that inflate small-sample bias, and (3) corroborating the direction of key effects in complementary analyses (unadjusted contrasts, time-to-event models, and landmark analyses). The primary objective of the analyses was associational (explanatory) inference, i.e., to quantify adjusted associations between pre-specified candidate factors and the odds of belonging to the EGF versus ULS group. However, we did not aim to develop or validate a clinical prediction model. Accordingly, measures such as AUC and a Youden-derived threshold are reported only to describe the apparent, within-sample discriminative performance of selected pre-specified landmark markers (e.g., 6-month creatinine) and should not be interpreted as externally validated decision thresholds. No internal or external validation, recalibration, or model updating was performed.

## Results

Baseline recipient characteristics were broadly comparable between groups (Table 1). The main between-group contrast was a shorter cold ischemia time (CIT) in the ULS, with the total ischemia time showing a concordant pattern. Preoperative cytotoxic antibody levels were low and similar in both groups.

**Table 1:** Baseline characteristics of kidney transplant recipients by outcome (mean ± SD; ranges shown where SD exceeds the mean).

Variable	ULS ≥ 25y	EGF ≤ 10y	*P*
Recipient age at transplantation (y)	31.6 ± 9.3	34.8 ± 11.1	0.093
Time from the onset of kidney disease to KTx (y)	10.9 ± 7.4	10.5 ± 8.2	0.530
Duration of HD (days)	815.5 ± 781.5	735.2 ± 599.8	0.867
Duration of peritoneal dialysis (days)	191.8 ± 205.7 (13–859)	147.8 ± 155.9 (2–530)	0.726
BMI recipients at the time of KTx	21.3 ± 3.0	21.3 ± 4.7	0.583
Warm ischemia time (min)	6.9 ± 10.5 (0–60)	3.7 ± 2.7	0.373
Anastomosis time (min)	28.5 ± 11.0	28.6 ± 7.3	0.432
Cold ischemia time (min)	1281.8 ± 473.9	1764.8 ± 564.9	0.016
Total ischemia time (min)	1318.3 ± 449.2	1799.1 ± 567.1	0.017
Maximum cytotoxic antibodies (%)	15.4 ± 22.5 (0–93)	16.1 ± 24.2 (0–96)	0.598
Minimum cytotoxic antibodies (%)	0.9 ± 2.9 (0–16)	1.5 ± 4.1 (0–30)	0.438
Preoperative cytotoxic antibodies (%)	1.4 ± 4.0 (0–20)	1.5 ± 3.8 (0–30)	0.682

Donor and procedural characteristics were broadly similar between groups among cases with available documentation (Table 2).

**Table 2:** Donor and procedural characteristics by outcome group.

Variable	ULS ≥ 25y	EGF ≤ 10y	*P*
Donor sex (F/M)	F 13/46 (28.3%), M 33/46 (71.7%)	F 19/53 (35.8%), M 34/53 (64.2%)	0.519
Donor–recipient sex concordant (yes = 1)	25/46 (54.3%)	33/53 (62.3%)	0.540
Transplanted kidney side (L/R)	L 10/19 (52.6%), R 9/19 (47.4%)	L 12/26 (46.2%), R 14/26 (53.8%)	0.668
Iliac fossa implanted (L/R)	L 20/49 (40.8%), R 29/49 (59.2%)	L 18/56 (32.1%), R 38/56 (67.9%)	0.330

Living-donor transplants were rare in both groups. Follow-up in ULS extended beyond 25 years by design, whereas EGF was defined as graft failure within 10 years. The distribution of recipient sex and primary kidney disease was broadly similar, with chronic glomerulonephritis predominating among cases with a documented etiology. Pre-transplant RRT patterns differed descriptively in the available records, but dialysis exposure (duration of hemodialysis and peritoneal dialysis) did not differ significantly between groups (Table 1). Peritoneal dialysis–related peritonitis occurred in both groups without a clear between-group signal. ABO compatibility was complete in both groups, except for one ULS pair with a missing donor blood type. Rh compatibility was documented in a similar proportion of pairs, and HLA matching categories were broadly comparable between groups (Table 3). Where documented, donor age and sex distributions were similar between groups, as were donor–recipient sex concordance and procedural descriptors such as graft laterality and implant site (Table 2). Early postoperative courses were broadly similar between groups, including rates of acute tubular necrosis and the need for post-transplant dialysis (Table 4). Within the first year, clinically recognized rejection was less frequent in ULS than in EGF, and the treatment indicators suggested a lower overall rejection burden in ULS (Table 5).

**Table 3:** Number of compatible donor-recipient pairs in the HLA system in the ULS and EGF groups.

HLA matching category	ULS ≥ 25y—N	EGF ≤ 10y—N
Incompatibility in HLA	2	2
1 matching pair in HLA	16	8
2 matching pairs in HLA	18	27
3 matching pairs in HLA	17	17
4 matching pairs in HLA	3	7
5 matching pairs in HLA	2	0
6 matching pairs in HLA	1	0

**Table 4:** Selected preoperative and postoperative complications or diseases in the study groups.

Variable	ULS ≥ 25y	EGF ≤ 10y
Hypertension before KTx	87.9%	98.4%
HBV infection before Ktx	32.2%	32.8%
Carbohydrate metabolism disorders before KTx	0	4.9%
Urinary tract infection as an early complication of KTx	44.1%	42.6%
Perinephric hematoma	23.7%	29.5%
Hypertension is present in the late period after KTx	92.9%	100%
Carbohydrate metabolism disorders is present in the late period after KTx	11.9%	27.9%
HBV infection is present in the late period after KTx	51%	57%
HCV infection is present in the late period after KTx	46%	46%

**Table 5:** Rejection-related outcomes within 1 year.

Variable	Threshold	ULS n/N (%)	EGF ≤ 10y n/N (%)	OR (ULS vs. EGF ≤ 10y)	*P*
Number of cycles of recognized rejection in the first year after KTx	≥1	20/59 (33.9%)	32/61 (52.5%)	0.46	0.045
Number of cycles of recognized rejection in the first year after KTx	≥2	4/59 (6.8%)	11/61 (18.0%)	0.33	0.096
Number of days of graft rejection treatment in the first year after KTx	≥1	24/59 (40.7%)	39/61 (63.9%)	0.39	0.017
Number of days of graft rejection treatment in the first year after KTx	≥3	23/59 (39.0%)	39/61 (63.9%)	0.36	0.010
Number of rejection treatment cycles in the first year after KTx	≥1	24/59 (40.7%)	39/61 (63.9%)	0.39	0.017
Number of antirejection treatment cycles in the first year after KTx	≥2	15/59 (25.4%)	23/61 (37.7%)	0.56	0.172

Longitudinal laboratory profiles favored ULS at later time points (Table 6). Although the creatinine level on postoperative day 1 was higher in the ULS group, serum creatinine levels at 6 months, 1 year, and 5 years were consistently lower in the ULS group, consistent with better, longer-term graft function. Hemoglobin was higher in the ULS group at 5 years, whereas leukocytes and platelets did not show meaningful between-group differences over time. Electrolytes were largely similar, with only small differences at isolated time points (Table 6).

**Table 6:** Selected blood tests in specified time periods after transplantation (Mean±SD; ranges shown where SD exceeds the mean).

Variable	ULS ≥ 25y	EGF ≤ 10y	*P*
HGB (g/dL)—1 day after KTx	10.1 ± 2.1	9.8 ± 1.9	0.595
HGB (g/dL)—1 month after KTx	10.2 ± 1.7	9.8 ± 2.1	0.309
HGB (g/dL)—6 months after KTx	13.5 ± 1.9	12.8 ± 3.0	0.214
HGB (g/dL)—1 year after KTx	13.4 ± 2.4	13.1 ± 3.2	0.602
HGB (g/dL)—5 years after KTx	13.1 ± 2.3	11.2 ± 2.4	<0.001
WBC (/μL)—1 day after KTx	7389.7 ± 2065.1	8291.3 ± 3241.9	0.125
WBC (/μL)—1 month after KTx	8117.4 ± 2946.3	7964.1 ± 3300.2	0.806
WBC (/μL)—6 months after KTx	7700.0 ± 2520.9	7830.5 ± 2599.9	0.790
WBC (/μL)—1 year after KTx	7337.8 ± 2040.2	7591.1 ± 2407.7	0.557
WBC (/μL)—5 years after KTx	8602.3 ± 9163.1 (4000–72,000)	6986.2 ± 1757.9 (2300–10,500)	0.202
PLT (/μL)—1 day after KTx	183880.6 ± 62195.2	195156.2 ± 69088.9	0.498
PLT (/μL)—1 month after KTx	219725.0 ± 68670.5	195532.4 ± 59506.7	0.122
PLT (/μL)—6 months after KTx	223119.0 ± 72636.7	220091.3 ± 90831.0	0.877
PLT (/μL)—1 year after KTx	213028.6 ± 72513.6	210360.8 ± 80752.9	0.879
PLT (/μL)—5 years after KTx	238816.7 ± 111514.6	214081.5 ± 70173.4	0.244
Creatinine (mg/dL)—1 day after KTx	9.8 ± 3.6	7.0 ± 2.1	0.031
Creatinine (mg/dL)—1 month after KTx	2.3 ± 2.4	2.6 ± 2.4	0.402
Creatinine (mg/dL)—6 months after KTx	1.3 ± 0.5	2.1 ± 1.7	<0.001
Creatinine (mg/dL)—1 year after KTx	1.3 ± 0.4	2.1 ± 1.2	<0.001
Creatinine (mg/dL)—5 years after KTx	1.6 ± 1.7	3.2 ± 2.7	0.004
Potassium (mmol/L)—1 day after KTx	4.5 ± 0.8	4.4 ± 0.8	0.457
Potassium (mmol/L)—1 month after KTx	4.5 ± 0.6	4.6 ± 0.7	0.270
Potassium (mmol/L)—6 months after KTx	4.3 ± 0.5	4.3 ± 0.6	0.853
Potassium (mmol/L)—1 year after KTx	4.3 ± 0.6	4.3 ± 0.5	1.000
Potassium (mmol/L)—5 years after KTx	4.2 ± 0.8	4.6 ± 0.6	0.033
Sodium (mmol/L)—1 day after KTx	135.1 ± 6.9	133.3 ± 5.8	0.223
Sodium (mmol/L)—1 month after KTx	140.2 ± 5.2	138.3 ± 6.7	0.120
Sodium (mmol/L)—6 months after KTx	140.1 ± 4.8	137.4 ± 6.6	0.020
Sodium (mmol/L)—1 year after KTx	139.3 ± 5.3	139.0 ± 5.3	0.824
Sodium (mmol/L)—5 years after KTx	141.6 ± 3.5	140.8 ± 4.1	0.353

At the last follow-up, the ULS recipients were older, whereas the EGF recipients experienced graft failure at a younger age. Chronic rejection was the leading cause of graft loss in EGF, with a smaller subset showing concurrent or recurrent glomerular disease, while acute rejection was the terminal event in a minority of cases. Other causes were infrequent.

The univariate analyses of the binary and nominal factors are summarized in Table 7. The denominators varied because of incomplete archival documentation. Two signals were notable: ULS more often lacked documented post-transplant carbohydrate metabolism disorders and more often had no documented/treated rejection within the first year, consistent with a lower early immunological and metabolic burden.

**Table 7:** Univariate analysis of binary and non-binary nominal factors in the analyzed groups.

Variable	ULS ≥ 25y	EGF ≤ 10y	OR (ULS/EGF)	95% CI	*P*
Hypertension before KTx	51/58 (87.9%)	60/61 (98.4%)	0.121	0.014–1.020	0.052
Hypertension after KTx	52/56 (92.9%)	61/61 (100.0%)	0.095	0.005–1.756	0.117
Absence of carbohydrate metabolism disorders after KTx	52/59 (88.1%)	44/61 (72.1%)	2.885	1.052–7.912	0.039
No documented/treated acute rejection within 1 year	39/59 (66.1%)	29/61 (47.5%)	2.15	1.03–4.50	0.045
Donor male (vs. female)	33/46 (71.7%)	34/53 (64.2%)	1.42	0.60–3.33	0.519
Sex match donor–recipient (yes)	25/46 (54.3%)	33/53 (62.3%)	0.72	0.32–1.61	0.540
Female kidney / male recipient	5/46 (10.9%)	10/53 (18.9%)	0.52	0.17–1.65	0.270
Male kidney / female recipient	16/46 (34.8%)	10/53 (18.9%)	2.29	0.91–5.74	0.076
Right kidney transplanted (vs. left)	9/19 (47.4%)	14/26 (53.8%)	0.77	0.24–2.48	0.668
Right iliac fossa (vs. left)	29/49 (59.2%)	38/56 (67.9%)	0.69	0.32–1.47	0.330

Recognized rejection denotes clinically documented and/or biopsy-supported rejection episodes captured in the medical record and/or accompanied by recorded antirejection therapy; protocol biopsies were not performed.

Consistent with Table 5, the ULS experienced fewer rejection-related events during the first post-transplant year. This was evident both for clinically recognized rejection episodes and for treatment-based indicators, supporting a lower first-year rejection burden in ULS (Table 5).

**Figure 1:**
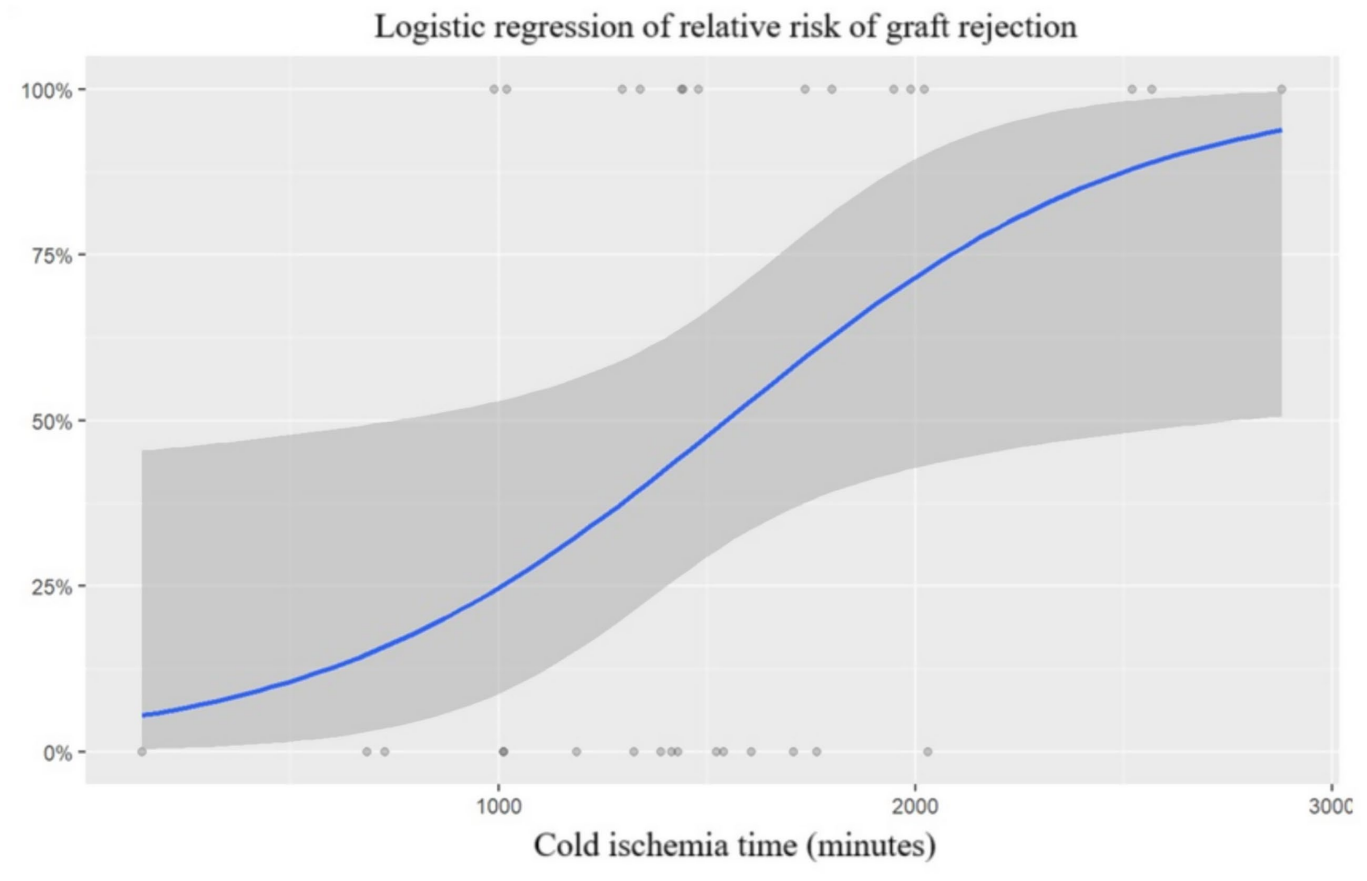
Exploratory univariable logistic regression: model-estimated probability of recognized acute rejection within 1 year by cold ischemia time (CIT).

Consistent with the baseline contrast, CIT differed between groups in univariate analyses. In an exploratory univariable logistic regression with recognized rejection within 1 year as the outcome, a longer CIT was associated with higher odds of recognized rejection (Figure 1). The fitted curve is presented to visualize the observed association and should not be interpreted as a validated prediction model.

### Multivariable analysis

In an unconditional logistic regression contrasting EGF (≤10 years) with ULS (≥25 years), CIT remained associated with higher odds of EGF versus ULS in adjusted analyses; however, covariate adjustment was constrained by archival missingness (complete-case subset). Therefore, the adjusted estimate is interpreted as supportive, alongside the unadjusted contrast and concordant time-to-event analyses. Recipient and donor ages were not significant. Linearity in the logit for the CIT was supported (likelihood-ratio test versus restricted cubic spline, *p* = 0.45). Because the total ischemia time was nearly perfectly collinear with the CIT (r = 0.999), it was not included in the same model. A sensitivity model substituting total ischemia for CIT (*n* = 82) showed a similar but attenuated, non-significant trend (OR 1.05 per 60 min; 95% CI 0.99–1.10). Attempts to adjust for transplant year (1980–1995) in the CIT subset led to separation and unstable estimates, which should be acknowledged as a limitation, and a potential period effect cannot be excluded (Table 8).

**Table 8:** Multivariable logistic regression—odds of EGF (≤10 years) vs ULS (≥25 years).

Variable	Adjusted OR	95% CI low	95% CI high	*P*	AUC
CIT (per 60 min)	1.2899	1.0215	1.6288	0.032	0.859
Recipient age (per 10 y)	1.1558	0.352	3.7957	0.811	
Donor age (per 1 y)	0.9865	0.899	1.0825	0.774	

Post-transplant laboratory measurements (HGB, leukocytes, platelets, sodium, potassium, and creatinine) were deliberately excluded from the primary model because they are post-treatment variables that may lie on the causal pathway and therefore risk introducing post-treatment bias. As a secondary, descriptive discrimination analysis, we conducted univariate landmark (time point) analyses to summarize the within-sample prognostic separation at specific follow-up times. Creatinine measured at 6 months showed clinically relevant univariable discrimination for EGF status in this dataset: the odds ratio per 1 mg/dL was 4.38 (*p* < 0.001) with an AUC of 0.739 (*n* = 119). A Youden-optimal threshold of 1.8 mg/dL yielded a sensitivity of 0.49 and a specificity of 0.91. In contrast, the creatinine level at 1 month did not provide useful discrimination (OR per 1 mg/dL: 1.07; *p* = 0.396; AUC 0.581; *n* = 118). Other routine laboratory measurements at 1 day, 1 month, or 6 months did not demonstrate clinically meaningful standalone discrimination in analogous univariate analyses. Overall, the primary multivariable association model focuses on CIT, while creatinine at 6 months is reported as an exploratory, sample-dependent prognostic discriminator at the landmark time point.

### Time-to-event analyses

In the 0–10-year window, a cause-specific hazard model (approximated with a person-time Poisson regression due to low event counts) showed that each additional 60 min of CIT was associated with a higher cause-specific hazard of graft failure: adjusted HR 1.11 (95% CI 1.01–1.23, *p* = 0.032; events *n* = 11; analysis-set *n* = 25) after adjustment for recipient and donor age. When additionally adjusting for the first-year rejection burden (0/1/≥2 antirejection treatment cycles), the CIT effect attenuated, and the rejection burden showed an adverse trend: ≥2 vs. 0: adjusted HR 8.29 (95% CI 0.93–74.01, *p* = 0.058); 1 vs. 0: adjusted HR 5.29 (95% CI 0.49–57.62, *p* = 0.172); CIT per 60 min: adjusted HR 1.07 (95% CI 0.98–1.17, *p* = 0.122; events *n* = 11; analysis set *n* = 24). In the 12-month landmark set (restricted to recipients with a functioning graft at 12 months and complete covariate data), no failures occurred between 1 and 10 years, precluding hazard estimation; descriptively, the creatinine level at 12 months remained lower among long-term survivors. Fine–Gray competing-risk checks yielded effect directions concordant with the cause-specific approach. The competing risk was death with a functioning graft; although small counts limited precise sHR estimation, subdistribution effects pointed in the same direction as the cause-specific analyses.

## Discussion

End-stage renal disease continues to increase, making the improvement of long-term outcomes after kidney transplantation a central clinical goal. Better late outcomes reduce return to dialysis, alleviate waitlist pressure, and improve quality of life while reducing costs (19). The distinct contribution of our study is its focus on ultra-long survivors (≥25 years) and on early, observable signals that can be operationalized for within-center audits and quality improvement, rather than reiterating broad, non-actionable associations.

A key constraint is the limited effective sample for multivariable adjustment owing to archival missingness. Therefore, we treat the adjusted estimates as supportive and emphasize consistency across complementary analyses (group contrasts, regression, and time-to-event approaches). Accordingly, the findings are presented as hypothesis-generating and suitable for a within-center audit, pending external validation. Although one-year outcomes after KTx have improved substantially, longer-term gains have been less consistent across studies, and late graft loss remains multifactorial (6, 19–23). Some cohorts report continued improvement in 10-year graft survival despite an increasingly complex recipient profile, whereas others emphasize that progress beyond the first decade has been modest and uneven (19–23). This tension in the literature supports a pragmatic focus on early, observable signals that may be amenable to center-level optimization. Contemporary ultra-long survivor reports (>20 years) further suggest that excellent late function clusters with specific donor and immunologic characteristics, yet detailed evidence beyond 20 years remains limited (7). Against this background, our dataset isolates a small set of actionable levers observable within the first post-transplant year that are enriched among ultra-long survivors.

Across analyses in our cohort, fewer first-year rejection events and a lower rejection-treatment burden were consistently associated with the ultra-long survivor phenotype. This aligns with prior work linking acute rejection—particularly later or more severe episodes—to worse long-term trajectories, with risks shaped by timing, persistence, and histologic phenotype (19, 24–26). Importantly, our rejection metrics reflect recognized and treated episodes captured in historical records rather than protocol-biopsy-based subclinical injury, which likely attenuates the measured associations. Overall, the direction of the effect is consistent with the concept that minimizing early immunologic injury is a plausible pathway toward better long-term graft durability. Cardiometabolic comorbidities (hypertension and PTDM) were highly prevalent and documentation-sensitive in this archival dataset and are therefore discussed primarily as long-term care targets and context variables, rather than as independent determinants of ultra-long graft survival based on our multivariable evidence. A minority of reports have suggested paradoxical associations in which early treated rejection correlates with better long-term outcomes, potentially reflecting treatment intensification and residual confounding rather than the protective effect of rejection itself (27). In our data, lower first-year rejection burden consistently characterized ULS; therefore, we interpret rejection primarily as an avoidable early injury signal and propose that simple tracking of 0/1/≥2 antirejection treatment cycles could serve as a feasible unit-level audit metric rather than a prescriptive clinical recommendation (9). Recent data from the latest era similarly link longer CIT, DGF, and immunologic contexts to early rejection risk and inferior early function, reinforcing the rationale for a first-year rejection-free pathway for prevention and rapid treatment.

Histocompatibility and immunosuppression are well-established determinants of graft outcomes. In our cohort, HLA matching is presented descriptively, but stable multivariable testing was not feasible because key covariates were incompletely captured, and complete-case restriction reduced the effective sample. Maintenance immunosuppression regimens varied from 1980 to 1995 and were not uniformly documented. Therefore, we avoid underpowered regimens or HLA comparisons and instead emphasize early, auditable signals (CIT, first-year rejection burden, and early graft function profiles) that warrant validation in contemporary multicenter datasets. In our study, the pre-transplant RRT modality and duration of HD/PD did not differ meaningfully between the groups in the available records, and any descriptive contrasts should not be over-interpreted. Prior literature on PD versus HD before KTx remains mixed; some studies report better patient survival among those treated with PD, while others find no consistent association with long-term graft outcomes (5, 28, 29). Given the descriptive nature of our data and limited statistical signals, we treat the RRT modality primarily as a context for education and logistics, rather than as a causal lever for ultra-long survival.

In this study, the key factor for long-term KTx outcomes was the shorter CIT. The literature includes many studies describing the relationship between CIT and transplanted kidney function. CIT is a modifiable pre-transplantation risk factor that affects graft survival. Debout et al. (30), in their research, demonstrated that every hour of CIT can impact both graft and patient survival after KTx. Hariharan et al. (19), in their work, state that reducing the severity of perfusion injury with colder ischemia time may contribute to improving long-term kidney survival outcomes in the case of deceased donors. Similarly, Rego et al. (16), in their conclusions, note that in many scientific studies, one of the main factors influencing long-term survival of recipients is a low CIT. The results of Dziewanowski et al. (31), who analyzed the outcomes of Polish patients over a long-term period of more than 15 years post-transplantation and a short-term period of less than 6 years, also indicate that CIT is a strong independent factor inversely correlated with long-term graft survival. Newer paired-kidney analyses confirm that even relatively small increments in CIT increase the risk of DGF and suggest that machine perfusion may mitigate CIT-related injury—evidence that supports our proposed CIT time-budget as a pragmatic, center-level quality target (8).

Cardiovascular disease is the dominant cause of late morbidity and mortality after KTx, and hypertension is highly prevalent in this population (17, 32). In our archival dataset, hypertension was common in both groups, and any between-group contrast was borderline and sensitive to documentation completeness; therefore, we treat it as descriptive rather than as evidence of an independent determinant of ultra-long graft survival. Nevertheless, prior studies suggest that higher blood pressure (BP) after transplantation is associated with inferior graft outcomes, and BP control remains a central long-term management target (33–37). Importantly, among ultra-long survivors, patient outcomes may become increasingly driven by cardiovascular comorbidity even when graft survival is favorable, reinforcing the need to integrate BP and cardiometabolic stewardship into long-term pathways (7). We also note the behavioral dimension of long-term graft success. Depressive and anxiety symptoms are common in kidney replacement therapy and can undermine adherence to immunosuppression and follow-up, with downstream consequences for graft and patient outcomes (38–41).

Post-transplant diabetes mellitus (PTDM) and related carbohydrate metabolism disorders are common after KTx and are associated with cardiovascular complications, graft loss, and mortality (11, 42). In our study, carbohydrate metabolism disorders were more frequent among early failures but did not consistently remain significant after parsimonious adjustment; accordingly, we treat this signal as hypothesis-generating and documentation-sensitive. Regardless, proactive screening, PTDM-conscious immunosuppression, and metabolic management remain reasonable vigilance strategies. These conclusions are consistent with recent pooled evidence indicating higher all-cause and cardiovascular mortality and increased graft failure risk in PTDM (11). Early graft function markers have long been linked to downstream outcomes, and several studies report that lower creatinine in the first year after KTx characterizes longer graft survival (21, 27, 43). In our cohort, the 6-month creatinine level showed the most useful within-sample prognostic separation, whereas the 1-month creatinine level and other routine laboratory measures did not meaningfully discriminate between outcomes. This supports treating 6- and 12-month creatinine levels as feasible auditable key performance indicators (KPIs) for within-center monitoring and hypothesis-generating risk stratification (12). More broadly, landmark or dynamic prognostic frameworks can help operationalize repeatedly measured markers in future prospective datasets (13). Beyond baseline determinants, 6-month creatinine functions as a practical landmark marker that can support within-center audits and hypothesis-generating follow-up prioritization. Recipients with higher 6-month creatinine levels had markedly greater odds of falling into the EGF ≤ 10 group, whereas 1-month creatinine levels and other routine laboratory measures did not significantly discriminate outcomes. This supports embedding 6- and 12-month creatinine levels as auditable quality indicators alongside CIT reduction and first-year rejection prevention.

### Limitations

This single-center, historical cohort from one transplantation era (1980–1995) improves internal consistency but limits the generalizability to contemporary practice and immunosuppression. The rarity of ≥25-year graft survival and the modest cohort size (ULS, *n* = 59; EGF, *n* = 61) limit precision for small effects and interaction testing. In this historical cohort, further expansion of the analyzable comparator arm was constrained by archival covariate completeness. Therefore, we used a maximally inclusive EGF comparator, applied a parsimonious pre-specified adjustment, and emphasized effect sizes and concordance across complementary analyses. Because the groups were not individually matched, residual confounding cannot be fully excluded despite restriction to a single center and conservative pre-specified adjustment. Incomplete historical documentation further curtailed the effective sample for multivariable analyses (e.g., the CIT model included 25 complete cases), necessitating complete-case analysis and introducing potential bias if missingness was not completely at random. Retrospective chart abstraction from decades-old records also varies in completeness and granularity (handwritten entries and lack of electronic systems). Some clinically relevant constructs, such as subclinical rejection and protocol biopsies, were not systematically captured; consequently, first-year rejection metrics reflect recognized and treated episodes only. Primary kidney disease labels reflect historical diagnostic practice; frequent use of chronic glomerulonephritis as an umbrella term and limited biopsy confirmation (8 ULS, 12 EGF) increases the possibility of misclassification, which is difficult to resolve *post hoc*. These factors could attenuate or obscure etiologic patterns.

By design, outcome-defined sampling (ULS ≥ 25 years vs. EGF ≤ 10 years) is vulnerable to selection bias (exclusion of >10–<25-year trajectories; inclusion governed by data completeness) and survivor bias (conditioning on long-term survival in ULS may over-represent resilient phenotypes). To mitigate these concerns, we complemented group contrasts with time-to-event analyses (0–10-year cause-specific models, a 12-month landmark among recipients with a functioning graft at the landmark, and Fine–Gray competing-risk checks) and confirmed robustness to the EGF threshold (≤8 and ≤12 years). Nevertheless, residual selection and survivor biases cannot be excluded.

Methodologically, period effects cannot be fully ruled out. Attempts to adjust for transplant year in the CIT model produced separation and unstable estimates, and total ischemia time was nearly perfectly collinear with CIT (r = 0.999), precluding concurrent inclusion; thus, residual confounding by unmeasured secular changes is possible. External validity is further constrained by the predominance of deceased-donor transplants (only three living donors) and center-specific logistics (procurement–transport–implantation workflows), which influence the CIT. These constraints motivate cautious interpretation and underscore the need for prospective multicenter validation with standardized data capture and predefined time-updated covariates. Finally, the archival nature of the dataset constrained incremental model-comparison analyses that could formally quantify the added value of covariates (e.g., likelihood-ratio comparisons of 1-year creatinine models with versus without hypertension) and similarly limited stable multivariable testing of histocompatibility and maintenance immunosuppression. Future work should validate these signals in multicenter cohorts, where a larger control pool and standardized covariate completeness allow more stable multivariable estimation.

## Conclusion

Cold ischemia time emerged as the most consistent and actionable early factor associated with ultra-long graft survival, supporting its prioritization as a modifiable, center-level quality target. Six-month serum creatinine levels may serve as a pragmatic landmark indicator for within-center audits; however, they should not be interpreted as a validated decision threshold. Accordingly, we propose an auditable framework centered on minimizing the CIT, preventing first-year immunologic injury, and routinely monitoring early kidney function markers (e.g., 6–12-month creatinine) to support long-term horizon care planning. Because ultra-long survivors consistently showed a lower first-year rejection burden, centers should emphasize prevention, timely diagnosis, and simple audit-friendly tracking of rejection events and treatment intensity.

Metabolic risk warrants proactive management through early screening and PTDM-conscious immunosuppression, whereas early biochemical markers (particularly creatinine in the first year) can help guide follow-up intensity within audit programs. Other baseline procedural descriptors showed no meaningful signal in this dataset and should not be prioritized over the core levers: minimizing CIT, preventing first-year rejection, and optimizing cardiometabolic risk.

## Data Availability

The raw data supporting the conclusions of this article will be made available by the authors, without undue reservation.
